# Oral Immunological Profile Impact on Local and Systemic Disease

**DOI:** 10.1155/2020/5273046

**Published:** 2020-05-21

**Authors:** Marco Cicciù, Tolga Tozum, Claudio Stacchi

**Affiliations:** ^1^Department of Biomedical and Dental Sciences and Morphological and Functional Imaging, Messina University, Messina 98100, Italy; ^2^Department of Periodontics, College of Dentistry, University of Illinois at Chicago, Chicago, Illinois, USA; ^3^Department of Medical, Surgical and Health Sciences, University of Trieste, Italy

Recently, the international literature has focused on the potential correlation between the systemic diseases and typical oral clinical conditions, which deeply influence a patient's oral health. In addition, research in recent years has increasingly highlighted on odontology data for general patient health assessment. Therefore, the presence of chronic inflammatory processes at the oral level may prevent some patients from following a particular therapeutic path, such as chemotherapy for the cancer patients.

In this context, many medical specialists are therefore turning to dentists to determine the absence of acute or chronic inflammatory conditions in patients' mouths. Oral health status is an indicator of overall body health, and this can help inform a medical specialist on the most appropriate type of therapy to implement for a patient. Moreover, patients who begin systemic therapy with bisphosphonate drugs are required to have a high oral hygiene level with no infections. The mechanism of action of bisphosphonate drugs means that they impair bone healing and modeling, which in turn leads to increased risk of jaw osteonecrosis following surgical dental procedures. Oral infection increases the risk of patients developing this condition. Regarding diabetes in particular, several published papers have demonstrated how control of periodontal health may be useful in managing the general health of patients. There are also correlations observed between changes in oral inflammatory cytokines and cardiovascular risk. Oncological pathologies, which are often accompanied by a pattern of alterations in gene expression, have also been reported to exhibit oral manifestations with alteration of the oral mucosa. Moreover, these pathologies can be located both at the oral cavity and in other body areas, and for this reason, the first dental visit is crucial to have quick diagnosis.

This special issue aimed to highlight the close relationship between systemic disease and oral pathologies and to investigate the connection between alterations in the oral immunological profile and systemic disease. Researchers were invited to submit original research articles and review articles in this field, with a focus on profiles of oral inflammatory cytokines and gene expression related to systemic and local pathologies. Submissions concerning acute, chronic, and oncological conditions were all welcomed. In order to cover state-of-the-art research and understanding in all relevant disciplines, submissions covering anatomical, histological, and biological features of the oral immunological profile of systemic disease patients, together with bioengineering and tissue engineering research, were all encouraged.

Victor M Martines et al. in their paper aimed to compare variations in quantified tumor necrosis factor-alpha (TNF-*α*) levels in patients with periodontitis stage 2 grade B (POD2B) and/or type 2 diabetes (T2D) and to identify any relationships between this cytokine and these diseases. Kruskal-Wallis tests was used to identify differences in TNF-*α* levels, LI, PD, BMI, BG, and HbA1c by group. Differences (*p* < 0.001) were found for LI, PD, BG, and HbA1c. A Spearman test was used to calculate possible correlations between TNF-*α* levels and LI or PD identified a weak but significant negative correlation of TNF-*α* with LI (Rho = −0199; *p* = 0.012), and a moderately positive correlation of LI with PD (Rho = 0.509; *p* < 0.001). The authors concluded how no variation was found between TNF-*α* levels and the presence of POD2B, POD2B/T2D, or T2D, suggesting the absence of any direct relationship between progression of these diseases and TNF-*α* levels. However, a correlation was present between low TNF-*α* concentrations and greater LI.

Diana Peniche Palma et al. compared levels of matrix metalloproteinase-9 (MMP-9) and myeloperoxidase (MPO) in gingival crevicular fluid (GCF) from subjects with controlled and noncontrolled type 2 diabetes mellitus (T2D), with and without stage 2 grade B periodontitis (POD2B) versus healthy (H) subjects. The authors found how the highest concentration of MMP-9 corresponded to the H group, while the lowest corresponded to the T2D controlled group. Regarding MPO levels, the highest levels were associated with the T2D controlled with POD2B group and the lowest with the T2D controlled group. Therefore, no apparent relationship between the elevation of MMP-9 and MPO levels was observed among subjects with T2D, with and without POD2B, compared to H subjects.

Colonna et al. tested in an animal model the nerve regeneration technique with a hypoallergenic acellular dermal matrix used to wrap the microsurgical neural suture and investigated how the application of suture material could influence the oral health. The authors stated how no correlation between the material applied and oral status was recorded and, moreover, how the histological and functional assessments showed a functional recovery of the injured nerve in the test groups, stressed by the results of the grasping tests and the meaningful increasing in fiber diameter and higher *g*-ratio. Moreover, a connective tissue cuff distinguishes the distal portion of the injured nerve. Considering the easy availability and handling of the material used in this study, we can conclude that this experimental technique can be considered as a valid alternative to protect nerves in nerve wrap surgery.

Cervino et al. performed a literature revision of the diabetes and oral health correlations. In their paper a comprehensive review of the literature was conducted according to PRISMA guidelines accessing the NCBI PubMed database. The authors conducted the search of articles in English language. The results of the last 10 years have been considered, which present useful information regarding the oral conditions. A total of 17 relevant studies were included in the review. The study evaluated only papers with specific inclusion criteria regarding oral health. The works initially taken into consideration were 782; subsequently applying the inclusion and exclusion criteria, there were 42 works. After a careful analysis of the work obtained by two academics that have worked separately, there have been 17 studies. The authors concluded how the psychological and psychosocial alterations, certainly present in these patients, are probably due to local and systemic alterations; this is confirmed by the correlation between oral health and quality of life reported by the patients [1–5].

The published papers raised great visibility and specially the one about diabetes and oral health quickly reached about 22 citations in Scopus.

*Marco Cicciù* *Tolga Tozum* *Claudio Stacchi*
